# Prevalence of Class 1 Integron and In Vitro Effect of Antibiotic Combinations of Multidrug-Resistant *Enterococcus* Species Recovered from the Aquatic Environment in the Eastern Cape Province, South Africa

**DOI:** 10.3390/ijms24032993

**Published:** 2023-02-03

**Authors:** Oluwaseun Ola Adeniji, Nolonwabo Nontongana, Anthony Ifeanyin Okoh

**Affiliations:** 1 SAMRC Microbial Water Quality Monitoring Centre, University of Fort Hare, Alice 5700, South Africa; 2 Applied and Environmental Microbiology Research Group (AEMREG), University of Fort Hare, Alice 5700, South Africa; 3 Department of Environmental Health Sciences, College of Health Sciences, University of Sharjah, Sharjah P.O. Box 27272, United Arab Emirates

**Keywords:** antimicrobial agents, integron, mobile genetic elements, antibiotic resistance, *Enterococcus*, combination therapy, rate of kill

## Abstract

Enterococci are regarded as a better indication of faecal pollution in freshwater and marine waters. Their levels in seawater are positively connected with swimming-related gastrointestinal disorders. This study used an *Enterococcus*-specific polymerase chain reaction (PCR) to characterize the isolates. Classes 1 and 2 integrons were examined for environmental *Enterococcus* isolates using a standard biological procedure. All strains were assessed against a panel of 12 antibiotics from various classes using disc diffusion methods. The microdilution method was used to work out the minimum inhibitory concentration (MIC) according to the CLSI guiding principles. The combination therapy of the resistant drugs was evaluated using a checkerboard assay and a time-dependent test for assessing their bactericidal or bacteriostatic activity. The gene diversity of the tested organisms was analyzed with the aid of Enterobacterial Repetitive Intergenic Consensus (ERIC) PCR. In total, 57 *Enterococcus* spp. environmental samples were recovered, in which *Enterococcus faecalis* (33.33%) and *Enterococcus faecium* (59.65%) were the dominant species. Resistance to linezolid, ciprofloxacin, erythromycin, gentamicin, vancomycin, rifampicin, and tetracycline was prevalent. Fifty (50) strains tested positive for class 1 integron, more frequent in *Enterococcus faecium* and *Enterococcus faecalis* isolates, with no gene cassette array discovered. A combination of gentamicin (MIC 4 µg/mL) with vancomycin (MIC 256 µg/mL) antibiotics against *Enterococcus faecalis* showed antibacterial activity. In contrast, the combination of ciprofloxacin (1 µg/mL) with Ampicillin (16 µg/mL) antibiotics against *Enterococcus faecalis* showed a bacteriostatic effect. The ERIC-PCR analysis pointed out that most of the assessed isolates have close genetic similarities.

## 1. Introduction

Integrons are natural recombination systems with the capacity for capturing, assembling, and conveying multiple gene cassettes encrypting antibiotic resistance (AR), which are frequently found incorporated into mobile genetics elements (MGEs) [1]. Those accountable for multidrug resistance (MDR) are of three classes and are grouped depending on the integrase genetic sequence they are carrying [2]. Class 1 integrons are observed in Gram-negative organisms and are linked to both an upsurge in and spreading of antibacterial MDR across the globe [2]. They comprise 5 and 3 conserved segments (CS). The 5 -CS consists of an *int*I gene encoding an integrase (*int*I1), *att*I- a recombination site and a promoter that is liable for the expression of the gene cassettes captured [3]. However, a gene conferring resistance to quaternary ammonium compounds (qacE1), *sul*1-a sulfonamide resistance gene, and an ORF (*orf*5) of the unknown function contains the 3-CS [3]. A variable region separates these segments, in which one or several gene cassettes are infixed.

Findings into AR in the aquatic milieu have primarily focused on microorganisms of faecal source as they serve as markers of contamination that could be linked with infectious disease and can transmit resistance at the crossing point of the host environment [4]. Enterococci are part of human and animal gut microflora; they exist widely in nature and are used as an important indicator of faecal pollution in water bodies [5,6]. However, the *Enterococcus* species, such as *Enterococcus faecalis* and *Enterococcus faecium,* are responsible for many healthcare-acquired diseases [7,8]. Over the past few decades, *E. faecium* has swiftly developed as a global healthcare-associated disease-causing organism by effectively adjusting to circumstances in a hospital setting and gaining resistance against glycopeptides [9,10]. In addition, MGEs are essential in the spreading and tenacity of AR in *E. faecalis* and *E. faecium*. Hence, enterococcal MGEs can transmit resistance determinants to other pathogenic organisms [11]. The isolation of vancomycin resistance Enterococci in clinical and ecological samples around the world and particularly in South Africa, constitutes a severe health problem [6,12,13].

Because of variations in an integron-integrase gene, various classes of integrons were detected in Gram-negative bacterial pathogens. Among them, the class 1 and 2 integrons are more occurring and prevalent. In the hospital setting, integrons may carry one or several gene cassettes, each of which encrypts resistance to a distinct drug [14]. Even though class 1 integrons are usually linked with Gram-negative organisms, they are also detected in Gram-positive organisms amongst Staphylococcus, *Aerococcus*, and *Corynebacterium* [15]. The initial report of an integron that carried a gene cassette encoding *aadA* was from *E. faecalis* [16]. Xu et al. [17] detected both class 1 and class 2 integrons in clinical enterococci in South China. Studies examining AMR in natural environments are important in detecting “environmental reservoirs of resistance” and promoting an understanding of the paths of transmission of these non-susceptible organisms. Hence, to have a better insight into the dissemination of ARGs in surface waters, this research was designed to evaluate the class 1 integron, the antibiotic sensitivity pattern of resistant *Enterococcus* species isolated from Kidd’s Beach in South Africa, and in vitro activity of the drug combinations. As much as we know, there are no reports on integrons in environmental enterococci in the Eastern Cape Province, South Africa.

## 2. Results

**Figure 1:**
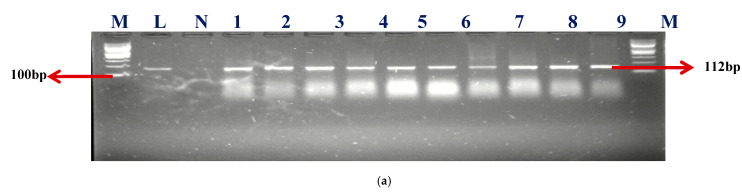

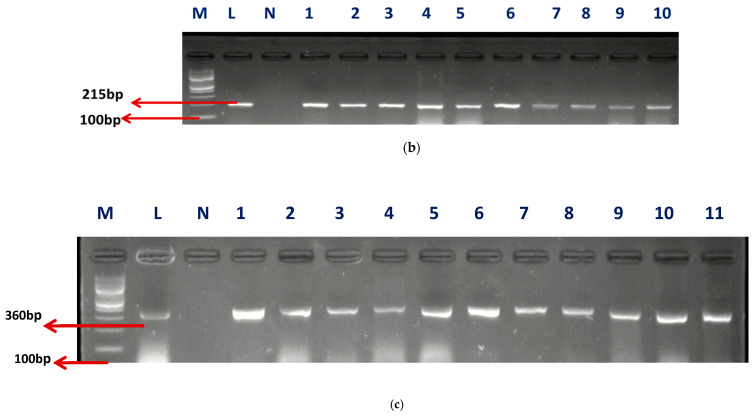
(**a**) The gel electrophoresis picture of identified Enterococcus genus at 112 bp. (**b**) The gel electrophoresis picture of *Enterococcus faecium* at 215 bp. (**c**) The gel electrophoresis picture of *Enterococcus faecalis* at 360 bp.

A sum of 57 isolates collected from AEMREG culture collection was identified as *Enterococcus* using Polymerase chain reaction (PCR) in agreement with our earlier study [8]. The identification of retrieved isolates was confirmed to be unchanged. Sixty percent (59.65%) of the isolates account for *Enterococcus faecium* (*E. faecium*), 33.33% were *Enterococcus faecalis* (*E. faecalis*), and 7.02% were unidentified. The Gel electrophoresis picture of the confirmed isolates and speciation are shown in Figure 1a–c.

M- DNA Ladder (100 BP), L- Positive control –DSM-20478- N- negative control, LANE 1–10 positive isolates.

M- DNA Ladder (100 BP), L- Positive control –DSM-20478- N- negative control, LANE 1–10 positive isolates. 

M- DNA Ladder (100 bp), L- “Positive control –DSM-20478- N- negative control, LANE 1–11 positive isolates”.

### 2.1. Analysis of Integrons

**Figure 2:**
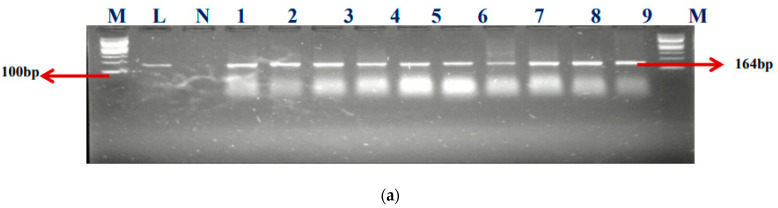

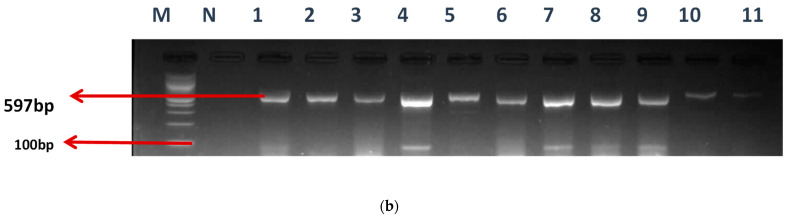
(**a**) The gel electrophoresis of amplicons (Expected band size 164 bp) of class 1 integron of *Enterococcus.* (**b**) The gel electrophoresis of amplicons (expected band size 597) of identified class 1 integron promoters of *Enterococcus*.

Among the total 57 enterococcal isolates screened for class 1 (*intI1*) and class 2 (*intI2*) integrons, 50 (87.7%) harbored class 1 integrons, but none was positive for class 2 integrons. Our findings showed that 50.88% (29/57) of *E. faecium*, 26.32% (15/57) of *E. faecalis,* and 6 (10.5%) of unidentified (UI) strains, respectively, were detected in the class 1 integrons. Clark et al. [18] documented the first discovery of the class 1 integron-related gene, *aadA*, found in *E. faecalis* strain W4470. The percentages of integron-positive and -negative isolates in this study were 87.5% (50/57) and 8.77% (5/57), accordingly. The electrophoresis image of the class I integron is presented in Figure 2a.

M- DNA ladder (100 bp), L- positive control –ATTCC 16066- N- negative control, Lane 1–9 positive isolates

### 2.2. Detection of Promoters in Class 1 Integron-Positive Enterococcus Strains

Various types of Pc variants have been identified in class 1 integrons based on their −35 and −10 hexamer sequences, and the relative strengths of their promoters have been verified by experimentation. The occurrence of these promoters was evaluated using a specific primer, as mentioned earlier. Twenty-seven (27) of the *E. faecium* isolates and eighteen (18) of the *E. faecalis* were found to have promoters. The gel electrophoresis picture is also shown in Figure 2b.

M-DNA ladder (100 bp), N- negative control, LANE 1–11 positive isolates

### 2.3. Antibiotics Susceptibility Profiles of the Enterococcus Species and Class 1 Integron

**Figure 3:**
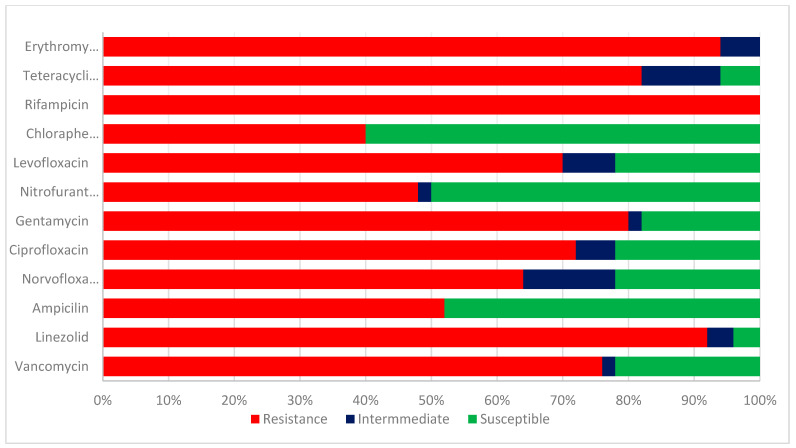
Antibiotic sensitivity pattern of class 1 integron *Enterococcus* species.

The test *enterococcus* species isolates were evaluated for their antibiotic sensitivities. The results of the antibiotic sensitivity pattern of class 1 integron *Enterococcus* species in Figure 3 are as follows: Ampicillin, 26 (52%); linezolid; 46 (92%); gentamicin; 40 (80%); Chloramphenicol; 20 (40%); Nitrofurantoin; 24 (48%); levofloxacin 35 (70%); ciprofloxacin, 36 (72%); norfloxacin 32 (64%); erythromycin, 47 (94%); vancomycin, 38 (76%); Rifampicin, 50 (100%); and tetracycline, 41 (82%). A higher percentage of resistance was detected in linezolid, erythromycin, rifampicin, vancomycin, and ampicillin. The isolates were susceptible to chloramphenicol (60%), nitrofurantoin (50%), and ampicillin (48%).

The chi-square was employed to compute the *p*-value in respect of resistant and susceptible figures of integron-positive and negative *Enterococcus* isolates. Integron occurrence was considerable (*p* < 0.05) in association with resistance against all the tested antibiotics, as represented in Table 1.

### 2.4. Evaluation of Minimal Inhibitory Concentration (MIC)

The values of the seven (7) antibiotics against the two major enterococcal strains are shown in Table 2. All isolates showed higher MIC values for erythromycin, vancomycin, linezolid, and rifampicin than the breakpoint (32 μg/mL, ≥128 μg/mL, >1042 μg/mL, and ≥32 μg/mL), respectively. Their breakpoint according to CLSI [18] is presented in supplementary Table S2. According to García-Solache and Rice [19], Enterococci are “considered to be susceptible to vancomycin, and are considered intrinsically resistant to clindamycin, quinupristin-dalfopristin, cephalosporins and aminoglycosides.” In agreement with this, the enterococci strains in the present study were quite resistant to aminoglycoside and beta-lactams but not susceptible to vancomycin. Enterococci are considered naturally non-susceptible to beta-lactam agents [20].

### 2.5. Checkerboard Assay

The FICI values of each tested strain (Table 3) demonstrated that gentamicin has a synergistic effect or additivity in combination with ampicillin, vancomycin respectively, against both *E faecalis* and *E faecium,* also ciprofloxacin showed synergy in combination with ampicillin against *E faecalis*. No synergistic effect was detected against all the tested strains with rifampicin when coupled with vancomycin, erythromycin, and ampicillin sequentially. A combination of Ampicillin with Ciprofloxacin and tetracycline showed an antagonistic effect, as shown in Table 3.

### 2.6. Time-Dependent Assessment of the Antibiotics Combinations against the Test Organisms

**Figure 4:**
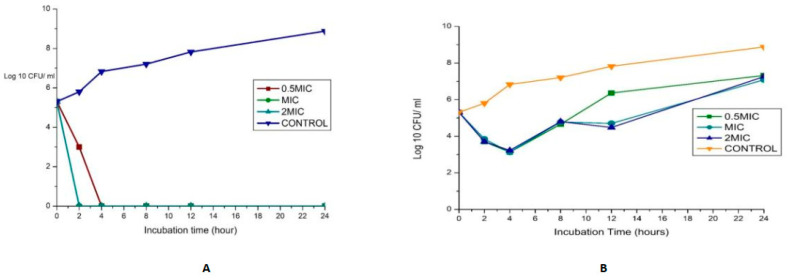

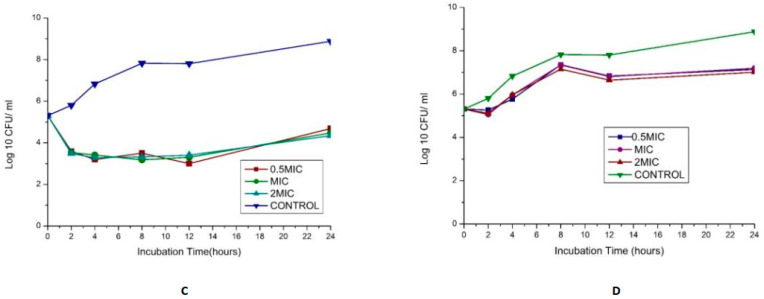
(**A**)Time-kill assay of a combination of gentamicin (4 µg/mL) with Vancomycin (128 µg/mL) antibiotics against *Enterococcus faecium_* bactericidal (**B**): Time-kill assay of the combination of gentamicin (4 µg/mL) with ampicillin (8 µg/mL) antibiotics against *Enterococcus faecium* _ bacteriostatic (**C**): Time-kill assay of the combination of gentamicin (4 µg/mL) with Vancomycin (256 µg/mL) antibiotics against *Enterococcus faecalis-* bacteriostati*c* (**D**): Time-kill assay of the combination of ciprofloxacin (1 µg/mL) with Ampicillin (16 µg/mL) antibiotics against *Enterococcus faecalis-* bacteriostatic.

The synergy of the studied organisms with ampicillin, gentamicin, and vancomycin was evaluated against *E. faecalis* and *E. faecium* and confirmed an FIC index <0.5 for every antibiotic combination. The results are displayed in Figure 4. In combination with gentamicin and vancomycin at a concentration of 4 and 128 µg/mL (MIC value of each drug), respectively, the original inoculum declined by 100% at about 2 h after treatment for MDR *E. faecium* at MIC and 2 MIC. This showed bactericidal activities against the pathogens. The bactericidal effect for 0.5 MIC was observed after 4 h, as shown in Figure 4A. This agrees with the findings of Cottagnoud et al. [21], who reported that gentamicin increases the efficacy of vancomycin against Penicillin-Resistant Pneumococci in the rabbit meningitis model.

The bactericidal action of gentamicin (4 µg/mL) coupled with ampicillin (8 µg/mL) against *E. faecium* showed bactericidal activities initially (≥3 log_10_ reduction in the size of the initial inoculum) after 4 h for 0.5 MIC, MIC, and 2 MIC, then a regrowth to a peak at 24 h, leading to a bacteriostatic effect (Figure 4B). In vitro studies showed that penicillin/ampicillin combined “with gentamicin is bactericidal against some vancomycin-resistant *E. faecium* isolates” that do not show any high-level gentamicin resistance (HLGR) [22,23]. The regrowth could be attributed to microorganism adaptation or drug degradation [24]. A combination of gentamicin (MIC 4 µg/mL) with vancomycin (MIC 256 µg/mL) antibiotics against *Enterococcus faecalis* showed antibacterial activity >3 log CFU/mL reduction of the initial inoculum. A combination of ciprofloxacin (1 µg/mL) with ampicillin (16 µg/mL) antibiotics against *Enterococcus faecalis* showed a bacteriostatic effect (Figure 4C,D).

### 2.7. Genetic Diversity of the Test Enterococcus Species

**Figure 5:**
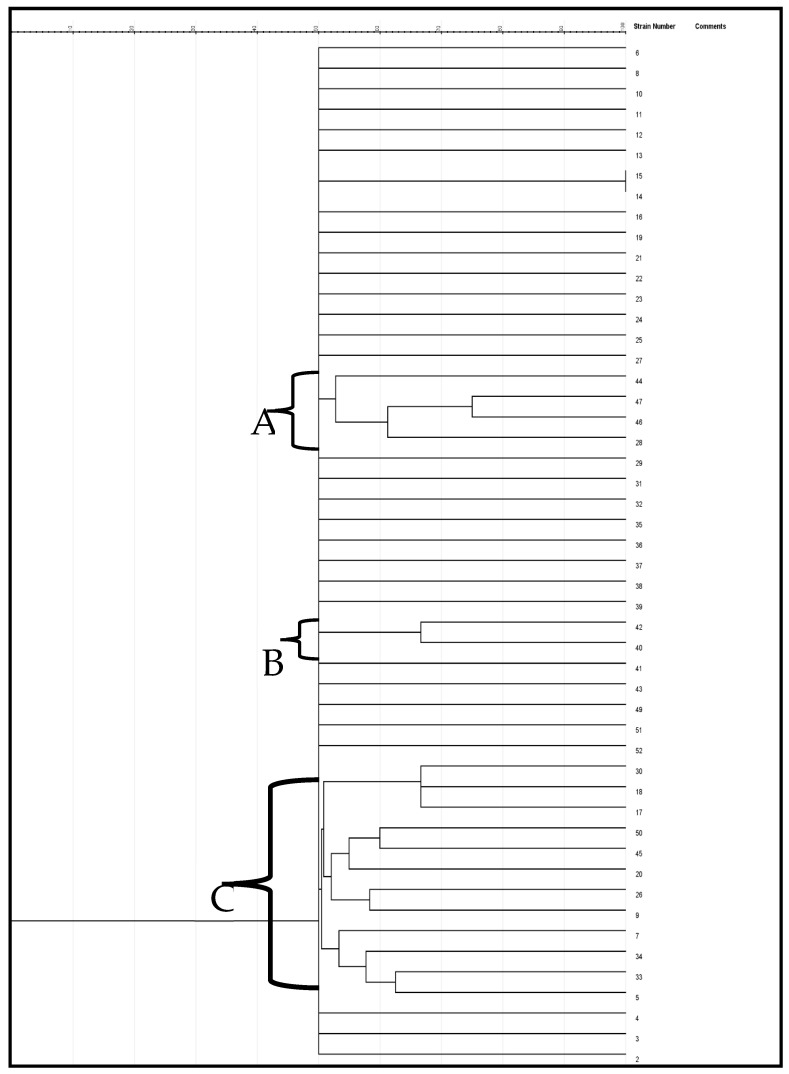
A neighbor-joining dendrogram of ERIC-PCR fingerprints of *Enterococcus* strains.

The genetic variation of the enterococcus species was evaluated using the ERIC-PCR test. Categorization of the genetic variability of the isolates by the dendrogram (Figure 5) was created via the jelJ software Logroño, 26004, Spain (version 2.0). The dendrogram was grouped into three clades (A, B and C) of *Enterococcus* species. Clusters that formed from the isolates in each group could suggest the source of the isolates.

## 3. Discussion

Enterococci are part of the gut bacteria of healthy humans and animals. They can find their route into the ecological systems such as surface water and soil via animal-human and feacal substances [25]. *Enterococcus faecalis* and *Enterococcus faecium* are major species of enterococcal used as feacal contamination indicators [26]; their existence is regarded as a marker of feacal pollution of environmental water sources [27]. These pollutions are usually caused by waste generated from homes and workshops, sewage from hotels and houses, and other related sources. In this study, two *Enterococcus* species, namely *E. faecalis* and *E. faecium,* were identified. The results obtained showed that *E. faecium* was one of the principal enterococci in water samples, suggesting a possible source of feacal pollution in the beach waters where the isolates were recovered from. Zhou et al. [28] reported *E. faecalis* and *E. faecium* as the main disease-causing organisms in humans among the enterococci in corroboration with the reports of Saingam et al. [29] and Alipour et al. [30].

Di Cesare et al. [31] and Vignaroli et al. [32] reported on *Enterococcus* that were non-susceptible to ampicillin, gentamicin, erythromycin, vancomycin, tetracycline, streptomycin, chloramphenicol, and ciprofloxacin in agreement with the findings of our current study. Numerous studies have revealed that enterococci in the aquatic milieu are non-susceptible to a range of antibiotics and show that antibiotic-resistant enterococci are not confined to clinical environments only but are also common in the environment [33,34]. Although vancomycin-resistant enterococci (VRE) are not often detected in unpolluted environments, discharged sewage from hospitals can be a source of these microorganisms in the water column [34,35,36]. In addition, it is significant that although in vitro antibiotic sensitivity testing should be used as a guide to examine microbiological susceptibility to different drugs, it does not often reflect the reality of the in vivo conditions [37].

A handful of studies about integrons in Gram-positive bacteria have been reported. The occurrence of class 1 integrons in Gram-positive organisms was first and foremost reported in *Enterococcus faecalis*, *Corynebacterium glutamicum*, and *Staphylococci* isolated from poultry litter [15,38], though, in China, the first extensive finding with clinical isolates was conducted [17]. Class 1 integrons were found in 15 *E. faecalis* and 29 *E. faecium* strains in this current study. Similar to our findings, Xu [17] detected class 1 integrons in 8 of the 10 enterococci isolates investigated. In addition, the report of Hajiahmadi et al. [39] concurs with our findings regarding the rate of recurrence of integrons, as classes 1 and 2 detected were reported as 86.7%, 6.7%, and 71.6%, 6% in clinical *E. faecium* and *E. faecalis* isolates, accordingly. No class 2 integrons were detected in all the isolates tested. They also identified 38.5% (10/26) and 23% (6/26) of their *enterococcus* isolates to be *E. faecium* and 27% (13/48) and 16.6% (8/48) to be *E. faecalis isolates*, having the *aadA1* and *dfrA7* gene cassettes, in that order. In this study, we did not find gene cassettes in the environmental enterococci isolates investigated.

In the present study, the selected enterococci strains showed higher MIC values than the breakpoint: erythromycin (32 μg/mL), gentamicin (>2 μg/mL), vancomycin (>128 μg/mL), linezolid (>1042 μg/mL), and Rifampicin (≥32 μg/mL). The common mechanisms of *Enterococcus* resistance to linezolid involve point mutations in chromosome 23S rRNA genes [40]. It also includes “plasmid-mediated chloramphenicol florfenicol resistance *cfr* gene or ribosome protection gene *optrA* and *poxtA* [41]. They may also develop increased resistance to penicillins by acquiring beta-lactamases or PBP4/5 mutations [42]. The principal resistant mechanisms of *E. faecium* to aminoglycoside entail “aminoglycoside-modifying enzymes (AMEs) including aminoglycoside nucleotidyltransferases (ANTs) aminoglycoside acetyltransferases (AACs) and aminoglycoside phosphotransferases (APHs) [43]. Vancomycin acts by binding to the D-alanyl-D-alanine (D-Ala-D-Ala) terminus and inhibits cell wall synthesis. Vancomycin-resistance genes are liable for substituting D-Ala-D-Ala with D-alanyl-D-lactate termini which results in a low binding affinity of vancomycin [44].

Combination treatment is suggested as an efficient way to fight against microbial resistance [45]. Clinical trials showed that patients who receive antimicrobial combination treatment could experience great clinical success and a reduced death rate [46]. At present, a wide range of synergy and efficient combinations against enterococcal diseases are being documented [47]. Baddour et al. [48] reported that beta-lactam antibiotics do not exhibit bactericidal effects against enterococci when administered alone, making treatment of systemic diseases difficult. Likewise, Baddour et al. [48] and Skinner et al. [49] reported synergy against all *E. faecium* and one *E. faecalis* vancomycin-resistant isolate by at least one antibacterial combination containing rifampicin. In the current investigation, a vancomycin plus rifampicin combination displayed an additive effect. Skinner et al. [49] documented that the definitive combination of a cell-wall-active agent (a beta-lactam or vancomycin) with an aminoglycoside leads to synergy against enterococci, which agrees quite well with our findings. Thus far, a vancomycin-based combination has demonstrated efficiency against resistant *Enterococcus* [49].

Bacterial non-susceptibility to antibiotics is correlated with a rise in MIC to more than one antibiotic; for instance, MICs of vancomycin and ampicillin against the MDR *Enterococcus* isolate assessed in this study were 256 µg/mL and 16 µg/mL, respectively. This implies that these antibiotics are no longer active at the determined tolerated dose. We have clearly shown that gentamicin and ciprofloxacin enhanced the effects of ampicillin and vancomycin against both *E. faecium* and *E. faecali*s, in which swift bactericidal action was observed. Aminoglycosides are effective, wide-spectrum drugs. Nevertheless, it is known that frequent exposure to aminoglycosides (gentamicin) raises the danger of nephrotoxicity if used in a systematic manner or ototoxicity, mainly affecting the vestibulocochlear system, and in turn, restricts its therapeutic usage [50].

ERIC-PCR is a commonly adopted PCR typing approach for analyzing genetic variation in organisms [51]. In the current study, the ERIC-PCR results presented a better overview of the *Enterococcus* species variety. The dendrogram generated by the computer-assisted analysis creating three clades is indicative of the high genetic variability of the beach water isolates. Moreover, they revealed that most of the appraised isolates have a close genetic affinity. Validation of genetic variability among *Enterococcus* isolates recovered from certain aquatic resources was also reported by Wei et al. [52]. The lack of ability of ERIC-PCR to type some strains is remarkable in this study. Ramazanzadeh et al. et al. [53] and Ekundayo & Okoh [54] discovered the incapacity of ERIC-PCR in typing some *E. coli* strains. Likewise, Prabhu et al. [55] reported that thirteen out of forty *E. coli* isolates were not typeable by ERIC-PCR, even as Ramazanzadeh et al. [53] also reported that twenty-five of two hundred and thirty *E. coli* isolates were not ERIC-PCR typeable.

## 4. Materials and Methods

### 4.1. Bacterial Isolation and Extraction of Genomic DNA

Enterococci species used in this study were collected from the culture collections of the Applied and Environmental Research Group (AEMREG) at the University of Fort Hare, South Africa, as part of our previous study [6]. A total of 57 isolates were recovered from glycerol stocks of the culture collection. The isolates were revived in Luria Bethani broth with a shaking incubator at 37 °C for 16–18 h. Thereafter, the genomic DNA of each MDR Enterococci used as a template for polymerase chain reaction (PCR) assays were extracted by the boiling method described by Garrido-Maestu et al. [56].

### 4.2. Detection of Tuf Gene for Confirmation of Enterococcus Genus and Enterococcus Speciation Using PCR

The identities of the presumed enterococci isolate, as described by Ke et al. [57], were then verified using PCR amplification of the *Enterococcus*-specific *tuf-*gene (amplicon size 112 bp). *Enterococcus faecalis* (DSM 20478) was used as a positive control). PCR amplification was carried out in a 25 μL reaction. The cycling conditions were as follows: initial denaturation at 94 °C for 3 min, followed by 30 cycles at 94 °C for 30 s, annealing at 53 °C for 45 s, extension at 72 °C for 60 s, and final extension step at 72 °C for 7 min. PCR products were separated by electrophoresis in a 2% agarose gel containing 5 μL of ethidium bromide (Sigma-Aldrich, St. Louis, MO, USA) at 100 V for 45 min in 0.5 TBE buffer and then were visualized with a UV transilluminator (ALLIANCE 4.7, A XD-79.WL/26MX, France). All PCR-confirmed enterococci isolates underwent the next round of PCR to identify their species. The list of primers with their amplicon size is presented in Supplementary Table S1.

### 4.3. Antibiotic Sensitivity Testing

Antibiotic sensitivity against a group of 12 antibiotics (Mast Diagnostics, Bootle L20 1EZ UK) was determined using the Kirby Bauer agar disk diffusion procedure as outlined in the Clinical and Laboratory Standard Institute (CLSI) guidelines [18]. The antibiotics selection was based on those used to treat infections caused by this organism. Each isolate was assessed against a panel of antibiotics including ampicillin (10 μg), vancomycin (30 μg), linezolid (30 μg), ciprofloxacin (5 μg), levofloxacin (5 μg), norfloxacin (10 μg), nitrofurantoin (300 μg), chloramphenicol (30 μg), tetracycline (30 μg), erythromycin (15 μg), gentamicin (10 µg) and rifampicin (5 μg). Consequently, the incubation of plates was carried out at 37 °C for 16 to 18 hrs. The antibiotics, except for vancomycin, were incubated for 24 h, followed by measurement of the zones of inhibition, with DSM 20478 used as a control.

### 4.4. Assessing the Genetic Variation of Enterococcus Species Isolates Using Enterobacterial Repetitive Intergenic Consensus Sequence PCR (ERIC-PCR)

The ERIC-PCR reactions were performed in 25 µL volumes of reaction. The primers ERIC1-FATGAAGCTCCTGGGGATTCAC and ERIC2-: AAGTAAGTGACTGGGGTGAGCG were used following the described method by Ateba & Mbewe [58]. Validation was achieved by resolving the PCR products in 3% agarose gel in a 5 x TBE buffer, followed by staining at 90 volts with ethidium bromide for 4 h. Then the images were visualized using an Ultraviolet (UV) transilluminator (Alliance 4.7, A XD-79.WL/26MX France). The clonal relatedness between *Enterococcus* species was digitized by GelJ version 2.0 Logroño, 26004, Spain (computer-assisted software analysis).

### 4.5. Molecular Evaluation of Class 1, 2, and 3 Integrons

The occurrence of *intI*1, *int*I2, and *int*I3 integrons in MDR Enterococci was examined by amplification of integrase genes using the specific primers (Table 4). The PCR mixtures were prepared in an aggregate volume of 25 μL, which was later amplified using a thermocycler. The electrophoresis of the amplicons was performed on 1.5% agarose gel and stained with ethidium bromide, followed by visualization as described above.

### 4.6. Characterization and Detection of Gene Cassette and Promoters

All integron–positive MDR *Enterococcus* strains were examined to detect the incidence of “internal cassettes genes by CS-PCR utilizing 3′CS and 5′CS primers”, as shown in Table 4. The description of the Pc-P2 region was conducted by amplifying and sequencing with the primers *int*I1F “(5=-CCTCCCGCACGATGATC-3=) and 5CSrevcompl (5=- CTTGCTGCTTGGATGCC-3=)” [63].

### 4.7. Evaluation of Minimal Inhibitory Concentration (MIC)

MIC testing was conducted based on the recommended CLSI method [18]. Briefly, solutions with varying concentrations of antibiotics were introduced into a sterile 96-well microtiter plate. The MICs for vancomycin were 1 to 512 µg/mL, 0.06 to 32 µg/mL for ampicillin, 0.125 to 64 µg/mL for erythromycin, 2 to 1024 µg/mL for linezolid, 0.06 to 32 µg/mL for rifampicin, 0.03 to 16 µg/mL for gentamicin and 0.015 to 8 µg/mL for ciprofloxacin. One hundred microliters (100 µL) of the highest concentration of the drug was transferred into each well of Column 1. Columns 2–10 contained only diluents, while Column 11 contained 100 µL of diluted standardized inoculum as growth control, and Column 12 contained 100 µL of the Muller Hilton broth (MHB) as sterility control. Antibiotics from columns 1–10 were mixed and transferred using a micropipette, producing fifty microliters of antibiotic per well (serial two-fold dilution). The standardized bacterial suspension was then diluted a hundred-fold in MHB. Thereafter, the adjusted bacterial suspension was added to each well containing different antibiotic concentrations as well as the control wells, yielding approximately 1 × 10^6^ CFU mL. The plates were then sealed, followed by incubation at 37 °C for 18–24 h. After incubation, thirty microliters of resazurin (0.015%) were added to all the wells and incubated further for 2–4 h for color change. The MICs were recorded as the concentration of antimicrobial agent that inhibits visible color change in broth medium wells. *Staphylococcus aureus* ATCC was used as reference organisms to validate the performance of each antibiotic stock solution. The reference strains were procured from the American Type Culture Collection (USA).

#### 4.7.1. Checkerboard Assay

The effects of antibiotic combinations were determined by the broth microdilution checkerboard technique described by Petersen et al. [64]. This was conducted in 96-well microtiter plates containing vancomycin and one of six other antibiotics (ciprofloxacin, ampicillin tetracycline, linezolid, rifampicin, and erythromycin). For each test bacteria isolate and antibiotic, the selected concentration ranges were dependent upon the earlier determined MICs. The antibiotic combinations are listed in Table S2. The checkerboard assay was performed in antibiotic combination assessment on two randomly selected isolates (MDR *E. faecium* and *E. faecalis).* The microplates were covered with the lid and incubated at 35 °C for 24 h. Antibiotic interactions were defined by the “fractional inhibitory concentration index (FICI)”. The ∑FICs (fractional inhibitory concentrations) were calculated as follows:

∑FIC = FIC A+ FIC B(1)

where FIC A is the MIC of antibiotic A in combination/MIC of antibiotic A alone, and FIC B is the MIC of antibiotic B in combination/MIC of antibiotic B alone. The combination is considered synergistic when the ∑FIC is ≤ 0.5, indifferent when the ∑FIC is > 0.5 to < 2, and antagonistic when the ∑FIC is ≥ 2 [65].

#### 4.7.2. Time-Kill Assay

Vancomycin, gentamicin, and ampicillin were utilized in the time-kill experiments. Each antibiotic was diluted to concentrations of 0.5 × MIC, 1 × MIC, and 2 × MIC [64]. The combined antibiotics (10 mL) each were tested in a 100 mL sterile flask. Mueller Hinton broth without test organisms was used as growth control. One hundred microliters of the adjusted 0.5 McFarland inoculum was then mixed with 10 mL of MHB to yield a final concentration of approximately 5 × 10^5^ CFU/mL. The cultures were incubated at 35 °C for 18 h with shaking at 120 rpm. Aliquots were taken from the cultures at 0, 2, 4, 8, 12, and 24 h. A 10-fold dilution series was carried out in sterile MHB, after which 100 µL of each suitable dilution was spread plated in triplicate on MHA plates. Parallel to each experiment, a growth control was performed. The mean colony counts (log CFU/mL) were plotted against the reaction times to produce the time-kill curves. The “bactericidal activity of single antibiotics or combinations was defined as ≥3 log_10_ CFU/mL decrease in the viable count compared with the initial inoculum [66]. Synergism and antagonism were respectively defined as ≥2 log_10_ CFU/mL decrease or increase in the viable count with the combination compared with the most active agent alone at different time points. [67].

### 4.8. Statistical Analysis

SPSS (version 21) (IBM, Chicago, IL, USA) was Employed for the Statistical Determinations in this Study. Connections between Antibiotic-Resistant Patterns and Integron-Positive Genotypes were Assessed using Fisher’s and Chi-Square Exact Tests. *p* < 0.05 was Regarded as being Significant Statistically.

## 5. Conclusions

The effect of hospital-acquired infections related to enterococci and the challenge of treating these diseases caused by resistance to antibiotics is problematic. The capability of enterococci to gain resistance genes via natural-conjugation gene transfer suggests that the presence of other resistances is a critical challenge. This study shows that the AR patterns of *Enterococcus* species recovered from the aquatic milieu and the occurrence of class 1 integron indicate the possibility of horizontal gene transfer of AR determinants in these strains. Even though the role of class 1 integrons is well reported in the spread of ARGs in Gram-negative organisms, little is known about Gram-positive. As far as we know, this research was the first that investigated the occurrence of integrons in environmental *Enterococcus* strains isolated from an aquatic resource in the Eastern Cape Province of South Africa. Increased incidence of vancomycin-resistant enterococci (VRE), ampicillin-resistant and linezolid-resistant enterococci in this study emphasized the need for intervention in the current antimicrobial management and monitoring. Despite the existence or absence of resistance genes within an integron, our results showed a considerate relation between the existence of integrons and reduced susceptibility to multiple antibiotics for enterococci. Therefore, there is a need to prevent the promotion of AR and the distribution of enterococci in aquatic resources by implementing necessary infection control measures and antibiotic stewardship programs.

## Figures and Tables

**Table 1:** A link between the antibiotic profiling and class 1 integrons in *Enterococcus* strains.

Antimicrobial Class	Antimicrobial Agents	Resistance (R)	Intermediate (I)	Susceptible (S)	Resistance (R)	Intermediate (I)	Susceptible (S)	Resistance (R)	Intermediate (I)	Susceptible (S)
		Integron-Positive Isolates n = 50			Integron-Negative Isolates n = 7			Total n = 57		
Aminoglycosides	Gentamicin	40 (80%)	1 (2%)	9 (18%)	6 (85.7%)	0	1 (14.3%)	46 (80.7%)	1 (1.8%)	10 (17.5%)
Tetracyclines	Tetracycline	41 (82.0%)	6 (12.0%)	3 (6.0%)	5 (71.4%)	0	2 (28.6%)	46 (80.7%)	6 (10.5%)	5 (8.8%)
Quinolones	Ciprofloxacin	36 (72%)	3 (6%)	11 (22.0%)	7 (100%)	0	0	43 (75.4%)	3 (5.3%)	11 (19.3%)
	Levofloxacin	35 (70%)	4 (8%)	11 (22.0%)	2 (28.6%)	0	5 (71.4%)	37 (64.9%)	4 (7%)	16 (28.1%)
	Norfloxacin	32 (64%)	7 (14%)	11 (22%)	2 (28.6%)	0	5 (71.4%)	34 (59.6%)	7 (12.3%)	16 (28.1%)
Penicillin	Ampicillin	26 (52%)	0	24 (48%)	4 (51.1%)	0	3 (42.9%)	30 (52.6%)	0	27 (47.4%)
Glycopeptide	Vancomycin	38 (76.0%)	1 (2.0%)	11 (22.0%)	5 (71.4%)	0	2 (28.6%)	43 (75.4%)	1 (1.8%)	13 (22.8%)
Macrolides	Erythromycin	47 (94%)	3 (6%)	0	7 (100%)	0	0	54 (94.7%)	3 (5.3%)	0
Phenicol	Chloramphenicol	20 (40%)	0	30 (60%)	1 (14.3%)	0	6 (85.7%)	21 (36.8%)	0	36 (63.2%)
Nitrofurantoin	Nitrofurantoin	24 (48%)	1 (2%)	25 (50%)	5 (71.4%)	0	2 (28.6%)	29 (50.9%)	1 (1.8%)	27 (47.4%)
Oxazolidinones	Linezolid	46 (92.0%)	2 (4.0%)	2 (4.0%)	5 (71.4%)	0	2 (28.6%)	51 (89.5%)	2 (3.5%)	4 (7.0%)
Ansamycins	Rifampicin	50 (100%)	0	0	7 (100%)	0	0	57 (100%)	0	0

**Table 2:** Minimum Inhibitory Concentration of the studied pathogens.

Pathogens	Vancomycin (µg/mL)	Ampicillin (µg/mL)	Erythromycin (µg/mL)	Linezolid (µg/mL)	Rifampicin (µg/mL)	Ciprofloxacin (µg/mL)	Gentamicin (µg/mL)
MDR *E. faecalis* (35)	256	16	2	2	2	1	4
MDR *E. faecalis* (41)	256	8	32	>1024	16	1	2
MDR *E. faecium* (26)	128	8	8	>1024	8	0.25	4
MDR *E. faecium* (44)	256	8	32	>1024	16	1	1

35, 41, 26, and 44 are isolate identification numbers.

**Table 3:** FIC values of the combined antibiotics for the test organisms.

Antibiotic Combination	MDR *Enterococcus Faecuim* (44) FIC	Type of Interaction	MDR *Enterococcus Faecium* (26) FIC	Type of Interaction
Vancomycin + Ampicillin	1	Additive	1	Additive
Ampicillin + Ciprofloxacin	2	Antagonistic	0.62	additive
Ampicillin + Gentamicin	0.75	additive	0.38	synergistic
Vancomycin + Gentamicin	0.625	Additive	0.375	synergistic
Tetracycline + Ampicillin	2	Antagonistic	2	Antagonistic
Rifampicin+ Vancomycin	1	Additive	2	Antagonistic
Rifampicin + Ampicillin	1.25	Indifference		
Erythromycin + ampicillin	1.5	Indifference		
Drug Combination	MDR *Enterococcus Faecalis* (41) FIC	Type of Interaction	MDR *Enterococcus Faecalis* (35) FIC	Type of Interaction
Vancomycin + Ampicillin	0.75	Additive	1	Additive
Ampicillin + Ciprofloxacin	0.53	Additive	0.155	Synergistic
Ampicillin + Gentamicin	0.75	Additive	0.75	Additive
Vancomycin + Gentamicin	0.75	Additive	0.27	Synergistic
Rifampicin + Ampicillin	1.5	Indifference	1.25	Indifference
Rifampicin + Vancomycin	1	Additive		
Erythromycin + ampicillin	1	Additive		

**Table 4:** List of primers used in this study for identification of classes of integron and internal gene cassette.

Target Gene	Primer Sequence (5′-3′)	Amplicon Size (Bp)	Reference
*IntI1*	F: CAG TGG ACA TAA GCC TGT TC R: CCC GAG GCA TAG ACT GTA	164	[59]
*IntI2*	F:TTATTGCTGGGATTAGGC R: ACGGCTACCCTCTGTTATC	232	[60]
*IntI3,*	F: AGTGGGTGGCGAATGAGTG R: TGTTCTTGTATCGGCAGGTG	600	[61]
*5’CS* *3’CS*	5′CS-F GGCATCCAAGCAGCAAG 3′CS-R AAGCAGACTTGACCTGA	variable	[62]
*attI2* *orfX*	attI2: GACGGCATGCACGATTTGTA orfX RGATGCCATCGCAAGTACGAG	varaible	[62]

## Data Availability

Not applicable.

## Supplementary Materials

The following supporting information can be downloaded at: https://www.mdpi.com/article/10.3390/ijms24032993/s1.
