# Supplementary material for: Prevalence of Class 1 Integron and In Vitro Effect of Antibiotic Combinations of Multidrug-Resistant Enterococcus Species Recovered from the Aquatic Environment in the Eastern Cape Province, South Africa
Source: Int J Mol Sci. 2023 Feb 3;24(3):2993. doi: 10.3390/ijms24032993 (PMC9917988; doi:10.3390/ijms24032993)
Supplement: Supplementary file 1 [file ijms-24-02993-s001.zip › ijms-2187637-supplementary.pdf]

SUPPLEMENTARY TABLE

Table S1: The list of primers with their amplicon size

**Target organisms**	**Target gene**	**Primer sequence (5′-3′)**	**PCR cycling conditions**	**Amplicon size (bp)**	**Reference**
E.faecalis ATCC19433		FL1 ACTTATGTGACTAACTTAACC<br>FL2 TAATGGTGAATCTTGGTTTGG	94 °C, 5 min; 35[94 °C, 30 sec; 52 °C,60 sec, 72 °C, 45 sec] 72 °C, 10 min	360	(Jackson *et al.,* 2004)
E.faecium ATCC19434		FM1 GAAAAAACAATAGAAGAATTAT<br>FM2 TGCTTTTTTGAATTCTTCTTTA	94 °C, 5 min; 30[94 °C, 30 sec;48°C, 60 sec; 72 °C, 40 sec] 72 °C, 10 min	215	(Jackson *et al.,* 2004)
Enterococcus ATCC 19433	*SodA*	F: TACTGACAAACCATTCATGATG<br>R: AACTTCGTCACCAACGCGAAC	94 °C,2 min, 30(94 °C 30 s, 55 °C , 55 s, 72 °C, 30 s, 72 °C for 4 min	112	Ke *et al.,* 1999

Table S2: List of antibiotics used and their breakpoints CLSI [20]

**Antibiotics(µg/ml)**	**susceptible(µg/ml)**	**Intermediate (µg/ml)**	**Resistance (µg/ml)**
Ampicillin	≤ 8	-	≥ 16
Vancomycin	≤ 4	8- 16	≥ 32
Ciprofloxacin	≤ 1	2	≥ 4
Gentamicin	≤ 4	8	≥ 16
Linezolid	≤ 2	4	≥ 8
Rifampicin	≤ 1	2	≥ 4
Erythromycin	≤ 0.5	1 - 4	≥ 8
